# Comparative Evaluation of Follicular Flushing Frequency and Scraping Time During Ovum Pick‐Up in Mares: Effects on Oocyte Recovery Rate and Technical Considerations

**DOI:** 10.1111/rda.70183

**Published:** 2026-02-12

**Authors:** Adrián Márquez‐Moya, Nerea Carreras‐Vico, Laura Sala‐Ayala, Rebeca Martínez‐Boví, Juan Cuervo‐Arango

**Affiliations:** ^1^ Equine Fertility Group, Facultad de Veterinaria Universidad Cardenal Herrera‐CEU, CEU Universities Alfara del Patriarca Spain

**Keywords:** equine reproduction, follicle, follicular flushing, horse, needle twisting, oocyte, TVA

## Abstract

Transvaginal ultrasound‐guided follicular aspiration or ovum pick‐up (OPU) has become the standard technique for oocyte collection in mares for intracytoplasmic sperm injection (ICSI). Although repeated follicular flushes and wall scraping are commonly used to improve oocyte recovery rate (ORR), the relative contribution of each remains unclear. This study aimed to compare the effects of multiple flushes versus controlled scraping time on ORR in mares. A controlled trial was conducted in two phases: (1) an ex vivo phase using slaughterhouse ovaries (*n* = 32), and (2) an in vivo phase in clinically healthy mares (*n* = 9). Follicles were assigned to two groups: multiple flushes (MF, 10 flushes with intermittent scraping, lasting 18 s in total) or single flush (SF, 18 s of continuous scraping followed by 1 flush). A total of 489 follicles were aspirated ex vivo and 143 in vivo. Results showed no significant difference in ORR between MF and SF groups in either phase (ex vivo: 63.8% vs. 59.5%; in vivo: 48.3% vs. 44.3%; *p* > 0.05). However, MF used significantly more flushing medium per follicle in the ex vivo model (*p* < 0.05). No significant differences were found in aspiration time or incidence of clots and blockages in the aspiration lines. A significant correlation was observed between clot number and aspiration system blockage (*r* = 0.497, *p* = 0.036). Continuous scraping without repeated flushing reduces medium usage but may increase operator fatigue and the risk of clot formation and system obstructions. Therefore, optimising scraping duration could improve the efficiency of oocyte recovery procedures while simplifying the technique and reducing costs. Further research is required to refine scraping protocols, reduce operator fatigue, and prevent complications related to clot formation.

## 1 Introduction

Transvaginal follicular aspiration, or ovum pick‐up (OPU), has been established as the technique of choice for obtaining immature oocytes in mares, intended for in vitro embryo production through intracytoplasmic sperm injection (ICSI) or in vitro fertilisation (IVF) (Felix et al. 2022). The use of OPU‐ICSI has markedly increased in recent years, driven by rising demand in parallel with advances in equine reproductive biotechnologies and the growing interest in preserving and multiplying the genetics of high‐value mares. Currently, it is considered the most efficient reproductive technique in terms of embryo per mare per year (Claes and Stout 2022; Cuervo‐Arango et al. 2019a; Lazzari et al. 2020).

The efficiency of in vitro embryo production largely depends on the performance of follicular aspiration, as recovery of a greater number of oocytes is positively correlated with the number of embryos generated per OPU‐ICSI session (Cuervo‐Arango et al. 2019b). One of the main indicators of OPU efficiency is the oocyte recovery rate (ORR, % recovered oocytes per follicle). Over its development, ORR in mares has shown highly variable values, ranging from initial reports of less than 30% (Brück et al. 1992) to current averages of approximately 50%–75% (Claes and Stout 2022; Cuervo‐Arango, Necchi, et al. 2025; Fonte et al. 2024; Lazzari et al. 2020).

At first, the technique was extrapolated from cattle without considering species‐specific anatomical and physiological differences in the attachment of the cumulus–oocyte complex to the follicular wall during the pre‐ovulatory period, which in mares markedly hinders oocyte release (Hawley et al. 1995). During the natural ovulation process, following the LH surge, increased hyaluronan synthesis causes the cumulus cells to expand, and the oocyte gradually detaches from the wall; however, since OPU procedures are usually performed before this physiological detachment is complete, the oocyte is still attached to the wall within a dense cellular and extracellular matrix (Gérard and Robin 2019). The current consensus is that the cumulus‐oophorus connection in mare oocytes is stronger than in other species and therefore it is often not possible to separate the oocyte from the wall by aspiration alone (Brück et al. 1999). Due to the mechanical resistance created by this attachment, additional techniques such as washing for hydrodynamic loosening and scraping for mechanical separation become necessary to release the oocyte.

Subsequently, several technical modifications were introduced to improve ORR in mares, including the use of double‐lumen needles, needle rotation during follicular application, specific aspiration and injection pressures, and the incorporation of repeated follicular flushing with heparinised medium (Colleoni et al. 2007; Cuervo‐Arango, Sala‐Ayala, et al. 2025). Unlike in cattle, where flushing is rarely necessary, in equine OPU it is thought to be critical to favour oocyte release. However, only ex vivo controlled studies have reported that performing MF increases ORR (Márquez‐Moya et al. 2025).

On the other hand, needle rotation during follicular flushing was incorporated under the assumption that it could facilitate detachment of the cumulus‐oocyte complex, based on postmortem studies reporting higher ORR (71%) when scraping the follicular wall with a bone curette (Alm et al. 1997) compared to only follicular fluid aspiration (31.2%). Experiments with postmortem ovaries showed that this manoeuvre significantly increases the likelihood of oocyte release by up to 12% (Márquez‐Moya et al. 2025), although in vivo evidence remains limited and often contradictory. Studies conducted by Cuervo‐Arango, Sala‐Ayala, et al. (2025) showed no difference in ORR between rotating or not rotating the needle. In a more recent study, the same author suggested that scraping performed by a single operator was not as efficient and attributed the higher ORR in two‐operator OPU procedures, compared to single operator, to more efficient follicle scraping (Cuervo‐Arango, Necchi, et al. 2025).

Nevertheless, an increased number of flushes is usually accompanied by longer scraping time, since the needle is rotated against the follicular wall during each flush, especially once the follicle has collapsed. This raises questions as to which of these two variables is primarily responsible for the observed improvement in ORR. In this context, it is necessary to evaluate in a controlled manner the relative effect of the number of flushes versus scraping time during equine OPU, to determine the exact influence of each variable, optimise the efficiency of the technique, and establish more practical and cost‐effective protocols.

Therefore, the main objective of this study was to compare the effect of the number of flushes versus follicular scraping time on the oocyte recovery rate in mares, and to describe the technical differences and potential complications associated with each approach.

## 2 Materials and Methods

### 2.1 Experimental Design

A controlled experimental approach was implemented in two phases: an ex vivo phase using slaughterhouse‐derived ovaries and an in vivo phase in live mares.

In both phases, standardised OPU protocols were applied, with two experimental groups: multiple flushes (MF) and single flush (SF), while maintaining the follicular wall scraping type constant. Both experimental techniques were tested simultaneously during several replicates. To establish a reference scraping time for 10 flushes, the procedure was initially timed: scraping was performed once the follicular wall collapsed during aspiration, with needle rotations of 90°–180°. A second operator recorded the total twisting (scraping) time between flushes, which averaged 1.8 s per flush, adding up a total scraping time of 18 s. Based on these measurements, the experimental groups were defined:

- MF: Each follicle was flushed 10 times, with scraping of the collapsed follicular wall between each flush, with an average scraping time of 1.8 s per follicle flush.
- SF: The follicle was aspirated and the wall scraped continuously for 18 s, followed by a single flush.

For the postmortem phase, ovaries were randomly assigned, alternating the order of replicates and ensuring equal numbers per session. In the in vivo phase, to avoid individual mare variation, each ovary within a mare was allocated to one of the groups, with left or right ovaries assigned to each group in alternate order.

The primary outcome measured was the oocyte recovery rate per aspirated follicle under the different technical conditions. Secondary parameters included aspiration time and medium volume used for all replicates. For aspiration time, a stopwatch was used to time the duration of the procedure from the puncture and aspiration of the first follicle to the last follicle of the ovary. To record the volume used, the flushing medium was placed in a graduated container (500 mL); the volume contained before washing the first follicle and the remaining volume after aspirating the last follicle in each replicate were noted. In live mares, additional data were recorded, including the number of times the aspiration system required flushing due to clogging and the presence of blood clots on the search dish. Clot counting was performed by registering the number of individual clots identified in the dish search (regardless of length and shape). A picture of each search dish was taken on every occasion to facilitate the clot counting.

### 2.2 Samples

#### 2.2.1 Ex Vivo Phase

A total of 32 excised ovaries were aspirated during March–April 2025, divided into two groups and nine replicates per group. They were obtained from a slaughterhouse located 45 min from the laboratory at CEU Cardenal Herrera University, Valencia, Spain. Ovaries were transported at ambient temperature (20°C–25°C) in plastic bags and processed within 6 h post‐mortem. The reproductive history, age, and breed of the mares were unknown.

#### 2.2.2 In Vivo Phase

Nine mares, aged 5–22 years and of different breeds (PRE = 3, Arabian = 3, crossbred = 2, Haflinger = 1), owned by the Teaching and Research Farm of CEU Cardenal Herrera University, Valencia, Spain, were enrolled in the study. All mares were clinically healthy and free of reproductive disorders that could interfere with oocyte collection. Antral follicle counts were performed by ultrasonography prior to aspiration, and each mare underwent a single OPU session in June 2025, after receiving approval from the Research Ethical Committee of the Universidad CEU Cardenal Herrera and by the regional official authority (Conselleria de Agricultura, Ganadería y Pesca; Licence ref. n. 2024‐VSC‐PEA‐0061).

### 2.3 Ovum Pick‐Up

In both phases, OPU was performed by a single operator as previously described (Sala‐Ayala et al. 2024).

A transvaginal ultrasound probe (Draminski Monoblock OPU guide, Sząbruk, Poland) connected to an ultrasound scanner (Draminski BLUE, Sząbruk, Poland) was used for follicular visualisation.

Follicles were aspirated and flushed under ultrasound guidance using a double‐lumen needle (Minitube, 12G × 25″, Minitube Iberica, Tarragona, Spain). The inner lumen was connected via silicone tubing to the aspiration port of a vacuum pump (Minitube aspiration and flushing pump for equine OPU, 230 V, Minitube Iberica, Tarragona, Spain), while the outer lumen was used for injection. The pump was set to 50 mmHg aspiration and 465 mmHg injection, corresponding to a flow rate of 0.8 mL/s at a fixed distance of 80 cm between the needle/ovary and the pump base, as flow rate varies with distance.

All visible antral follicles were punctured and subsequently flushed (according to treatment group). An assistant documented each procedure.

Lactated Ringer's solution supplemented with sodium heparin (Heparina sódica Sala 5000 IU/mL, Laboratorio Reig Jofre SA, Barcelona, Spain) was used as flushing medium (5 IU/mL). For each flush, enough medium was injected to re‐expand the follicle to its original size, using the pump's injection and aspiration pedals. Follicular fluid and flushing medium were collected in a 500 mL flask.

After all follicles within a replicate were aspirated, the system was thoroughly rinsed before removing the collection flask. A new sterile flask was placed before initiating the next replicate.

#### 2.3.1 Ex Vivo Procedure

OPU was performed directly through the ovarian surface without removing the tunica albuginea. Each ovary was stabilised against the ultrasound probe with the operator's right hand, while the left hand supported the probe handle and guided the needle using the index finger and thumb.

#### 2.3.2 In Vivo Procedure

For in vivo OPU, mares were premedicated with flunixin meglumine (Nixyvet 50 mg/mL, Divasa‐Farmavic S.A, Barcelona, Spain) 1.1 mg/kg IV. No prophylactic antibiotics were administered.

Sedation was induced using detomidine (Domidine, 10 mg/kg, Dechra, Bladel, Netherlands) and butorphanol (Butomidor, 10 mg/kg, Richter Pharma, Wels, Austria) each at 0.01 mg/kg IV. Boluses were repeated as needed to maintain an adequate anaesthetic plane throughout the procedure.

Before OPU, the rectum was manually emptied, the vagina was aseptically prepared, and a Foley catheter (22 FR) was placed into the bladder. The operator introduced the ultrasound probe transvaginally and, as in the postmortem protocol, manipulated both probe and needle with the left hand. In this case, the ovary was grabbed and fixed per rectum with the right hand. At the onset of aspiration, scopolamine butylbromide (Buscapina compositum, 4 mg/mL, Boehringer Ingelheim, Ingelheim/Rhein, Germany) (0.08 mg/kg) was administered IV, with additional doses given at the operator's discretion depending on rectal relaxation.

When switching to the contralateral ovary, the system was thoroughly rinsed and the collection flask replaced. The system was also flushed whenever deemed necessary by the operator (e.g., in the presence of blood or obstruction). All events were recorded by an assistant.

### 2.4 Oocyte Search

The operator responsible for oocyte retrieval was blinded to treatment groups. After each replicate, aspirated fluid was filtered through a nylon mesh (75 μm; EmCon filter, Kansas, USA). The retained material was rinsed with the same flushing medium until the effluent was clear. The filter content was then transferred to a Petri dish, rinsed with 20 mL of medium using a 20 mL syringe and 21G needle, and examined under a stereomicroscope (Stemi 508, Zeiss, Madrid, Spain).

For in vivo samples, the number of blood clots retained on the filter was recorded, and clots were placed in a separate Petri dish to facilitate oocyte search.

### 2.5 Statistical Analysis

All evaluated parameters (oocyte recovery rate, volume per follicle, time per follicle, blockage per ovary, flushes per ovary, and clots per ovary) were tested for normality using the Shapiro–Wilk test.

Since all datasets except blockage per ovary were normally distributed, unpaired Student's *t*‐tests were applied to compare mean replicate values between groups for the normally distributed variables, while a Mann–Whitney U test was used for blockage per ovary.

Additionally, a chi‐square test was used to assess differences in overall oocyte recovery per follicle.

Finally, Spearman's ranked correlation analysis was performed to evaluate the association between clot formation observed in the Petri dish and aspiration system blockage.

Data were presented as the mean ± standard deviation (SD). Statistical analyses were performed using IBM SPSS software, version 29.0 (IBM Corporation, New York, NY, USA). Statistical significance was considered when *p* < 0.05.

## 3 Results

### 3.1 Ex Vivo

A total of 9 replicates per group were performed, with 16 ovaries aspirated in each group. The mean ORR per replicate was 63.8% ± 11.4% in the MF group and 59.5% ± 11.6% in the SF group, with no significant difference between treatments (*p* > 0.05). Similarly, the overall number of oocytes recovered per follicle was 154/241 (0.64) for MF and 149/248 (0.60) for SF, with no significant differences (*p* > 0.05) (Table 1).

**TABLE 1:** Effect of the number of follicular flushes, with equal needle twisting time for follicular scraping, on oocyte recovery rate (ORR), oocytes per follicle, flushing volume per follicle, and aspiration time per follicle during follicular aspiration in ex vivo ovaries and in vivo mares.

Group	Replicates	ORR (%)	Oocyte per follicle	Volume per follicle (mL)	Time per follicle (min)
Ex Vivo					
MF	9	63.8 ± 11.4 (0.41–0.74)	154/241 (0.64)	11.5 ± 1.59^a^ (8.3–13.2)	1.03 ± 0.11 (0.9–1.2)
SF	9	59.5 ± 11.6 (0.42–0.76)	149/248 (0.60)	2.6 ± 1.02^b^ (1.6–4.4)	0.96 ± 0.26 (0.4–1.5)
In Vivo					
MF	9	48.4 ± 23.6 (0.14–0.83)	32/70 (0.46)	24.3 ± 11.6 (12.5–42.9)	2.2 ± 0.74 (0.9–3.3)
SF	9	44.3 ± 13.8 (0.20–0.63)	35/73 (0.48)	16.3 ± 6.6 (7.5–25)	2.1 ± 0.89 (1.1–3.3)

*Note:* The experimental groups for both ex vivo and in vivo trials were: 10 flushes (MF) and a single flush (SF), with an equal needle twisting time for follicular scraping. Within columns, different superscripts (a, b) denote statistically significant differences (*p* < 0.05). Data are presented as mean ± standard deviation; values in parentheses represent the minimum–maximum values, except for oocyte per follicle, where parentheses indicate the oocyte/follicle proportion.

In contrast, the flushing medium volume used per follicle and per oocyte was significantly higher in the MF group (mean volume per follicle 11.5 ± 1.59 mL vs. 2.6 ± 1.02 mL) (Table 1).

The mean time required to aspirate a follicle did not differ significantly between groups, averaging 1.03 ± 0.11 min in MF and 0.96 ± 0.26 min in SF (Table 1).

### 3.2 In Vivo

Nine mares were aspirated, resulting in 9 ovaries per treatment group. The mean ORR was 48.3% ± 23.6% in the MF group and 44.3% ± 13.8% in the SF group, with no significant differences (*p* > 0.05). The overall oocyte yield per follicle was 32/70 (0.46) for MF and 35/73 (0.48) for SF, also with no significant differences (*p* > 0.05) (Table 1).

No significant differences were found between the MF and SF groups in the volume of flushing medium per follicle (24.3 ± 11.6 vs. 16.3 ± 6.6 mL/follicle), the number of aspiration system blockages per ovary (0.78 ± 0.83 vs. 1.56 ± 2.01), or the number of rinses required per ovary (2.4 ± 1.0 vs. 3.1 ± 1.6) (*p* > 0.05) (Tables 1 and 2). Likewise, aspiration time per follicle was comparable between groups (2.2 ± 0.7, MF vs. 2.1 ± 0.86 min, SF) (Table 2).

**TABLE 2:** Effect of the number of follicular flushes, with equal needle twisting time for follicular scraping, on the number of aspiration system blockages and rinses, as well as the number of blood clots observed in the search dish, during follicular aspiration in live mares.

Group	Replicates (ovary)	Rinse per ovary	Blockage per ovary	Blood clots per ovary
In Vivo				
MF	9	2.4 ± 1.0 (1–4)	0.78 ± 0.83 (0–2)	6.4 ± 5.6 (0–15)
SF	9	3.1 ± 1.6 (1–6)	1.56 ± 2.01 (0–5)	11.2 ± 8.1 (2–28)

*Note:* For the in vivo experiment, the two groups are represented as follows: 10 flushes (MF) and a single flush (SF), with an equal needle twisting time for follicular scraping. Data are presented as mean ± standard deviation; values in parentheses represent the minimum–maximum values.

Finally, the number of blood clots recovered per replicate did not differ significantly between groups (6.4 ± 5.6, MF vs. 11.2 ± 8.1, SF; *p* > 0.05) (Table 2).

**FIGURE 1:**
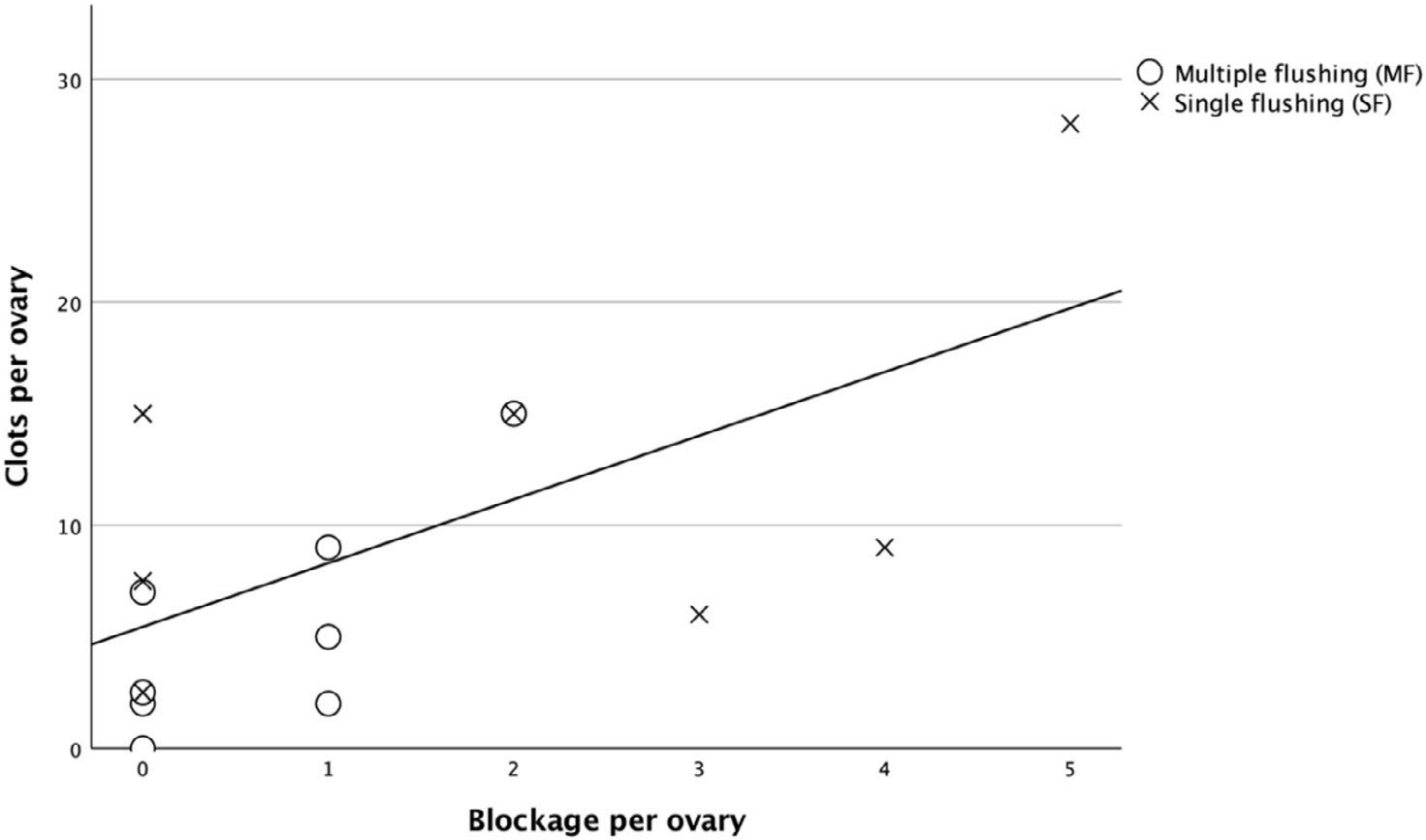
Scatter plot showing the positive relationship between Blockage (in the aspiration system during follicular aspiration) and clots (in the petri dish during searching). Each point represents an individual observation. The solid line indicates the linear regression fit, reflecting a moderate positive correlation (*r* = 0.497, *p* = 0.036).

A Spearman correlation analysis was performed to evaluate the relationship between clot formation and aspiration system blockage (Figure 1). The results revealed a moderate positive correlation (*r* = 0.497), which was statistically significant (*p* = 0.036, two‐tailed), suggesting that a higher number of clots was associated with an increased frequency of aspiration system blockage (Figure 1).

**FIGURE 2:**
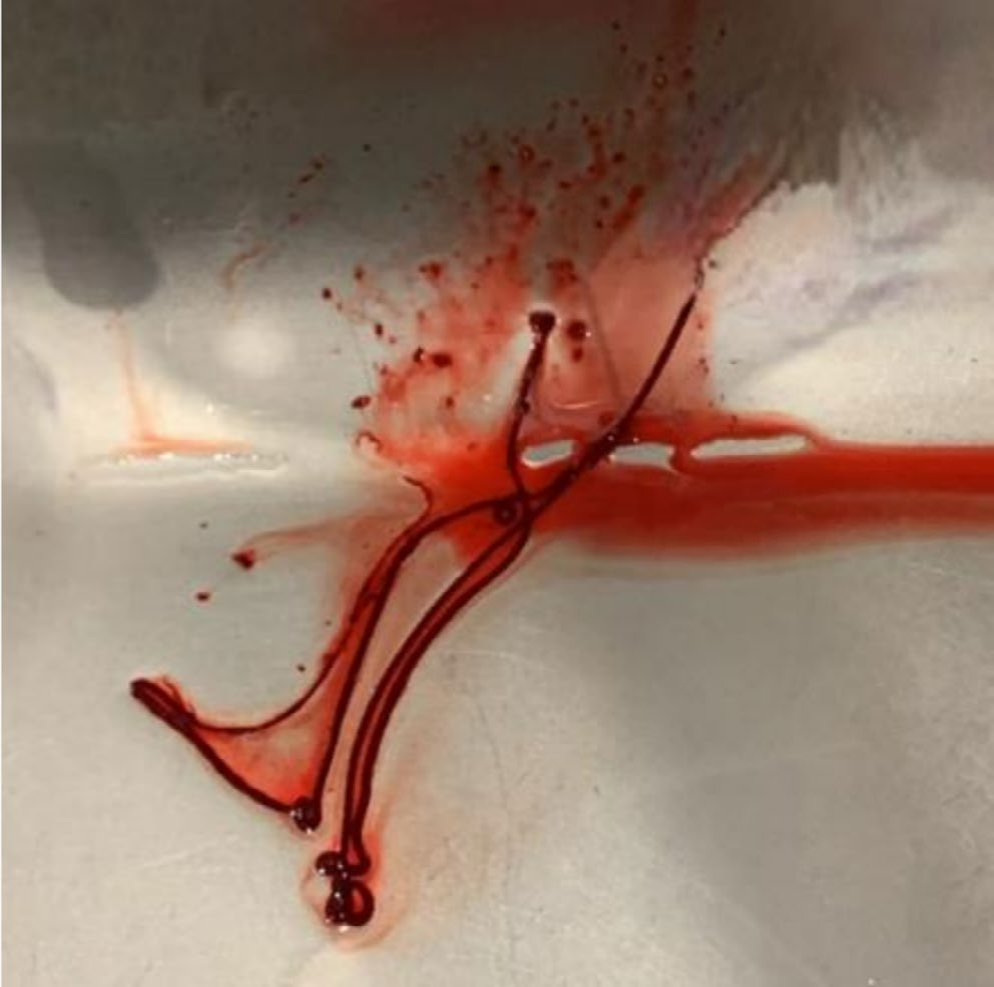
Spaghetti‐like clot formed within the inner needle (16G) resulting in system blockage.

**FIGURE 3:**
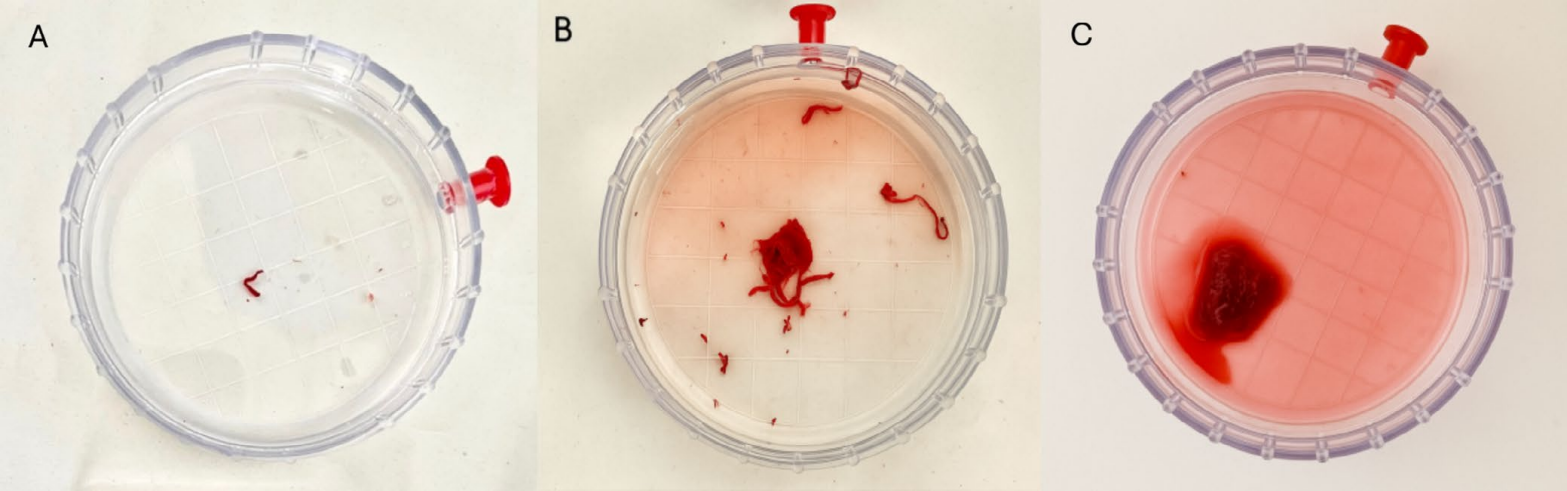
Oocyte search dishes (A–C) showing variation in the number of blood clots retained after filtration of the fluid collected during follicular aspiration and the associated discoloration of the medium. (A) Search dish with only one small clot; clear medium. (B) Search dish containing several small, spaghetti‐like clots (adopting the shape of the aspiration tubing) and slight reddish discoloration of the medium. (C) Large clot formed by aggregation of multiple spaghetti‐like clots; marked reddish discoloration of the medium.

During filtration, the clots frequently adopted the morphology of the tubing system, displaying elongated, spaghetti‐like structures (Figure 2). In addition, the clots present in the search dish often released erythrocytes into the medium, giving it a reddish hue that impaired visibility during oocyte searching (Figure 3). No oocytes were found in the dish from which the clots had been removed.

## 4 Discussion

In this study, a preliminary ex vivo experiment was conducted to comply with the three fundamental principles of animal experimentation: reduction, refinement, and replacement (Russell and Burch 1959). Although the ex vivo model has proven reliable for analysing factors influencing oocyte recovery rate (ORR) (Sala‐Ayala et al. 2024), full replacement was not feasible in this study. Parameters such as mare movement and the presence of blood or clots in the aspiration system could only be evaluated in vivo.

The in vivo results were consistent with those obtained in the ex vivo system, supporting the validity of the postmortem OPU simulator as a tool for analysing variables affecting ORR (Sala‐Ayala et al. 2024). The slight discrepancies observed between ORR in the two studies may be related to operator experience. Although the same operator performed all procedures, their experience in the in vivo technique was substantially lower. Furthermore, the overall mean ORR obtained (46%) was slightly below values reported in similar geographic regions for a single operator (54%) (Cuervo‐Arango, Sala‐Ayala, et al. 2025; Cuervo‐Arango, Necchi, et al. 2025), corroborating previous evidence of the influence of operator expertise on ORR (Claes and Stout 2022; Rodriguez et al. 2021). The ex vivo simulator enhances skills in handling the ultrasound probe, needle, and aspiration pump (Márquez‐Moya et al. 2025; Sala‐Ayala et al. 2024), however, the in vivo technique introduces additional challenges, including transrectal ovarian stabilisation, interference from animal movement, and the risk of trauma associated with excessive manipulation (Van den Branden et al. 2025; Cuervo‐Arango, Necchi, et al. 2025; Hinrichs et al. 2025).

It is well established that the number of antral follicles (AF) available and aspirated correlates positively with the number of oocytes recovered and overall success in OPU‐ICSI sessions (Cuervo‐Arango et al. 2019b; Breton and Lewis 2024; Scarlet et al. 2025). In this study, the number of aspirated follicles was lower than previously reported in Europe, with 15.9 follicles per mare compared to 25.9 in Claes and Stout (2022) and 24.5 for a single operator in Cuervo‐Arango, Necchi, et al. (2025), due to the limited number of animals and restricted experimental time, selection of mares based on AF count was not possible. Moreover, up to 30% of the mares were older than 20 years, which reduces total follicle counts (Cuervo‐Arango et al. 2019b; Fonte et al. 2024).

The present findings indicate that the duration of follicular scraping exerts a greater impact on ORR than the number of flushes. Previous ex vivo studies reported a positive association between increasing the number of flushes and ORR (Márquez‐Moya et al. 2025); however, in those studies, increased flushes were accompanied by longer scraping times, suggesting that improvements in ORR may be attributable primarily to scraping rather than flushing, consistent with our results.

The efficiency of follicular scraping has been debated. Its use in OPU originates from postmortem studies, showing a marked increase in oocyte recovery when follicles were scraped with a bone curette compared to syringe aspiration alone (31% vs. 71%) (Alm et al. 1997). Subsequent ex vivo studies demonstrated that scraping alone could improve ORR by up to 12% (Márquez‐Moya et al. 2025). However, in vivo studies have not reported significant differences in ORR when performing 10 flushes with or without scraping (Cuervo‐Arango, Sala‐Ayala, et al. 2025).

These findings highlight the controversy regarding the true contribution of the number of flushes and scraping to ORR. The apparent discrepancies may reflect a combined, non‐additive effect of both variables. In previous studies (Cuervo‐Arango, Sala‐Ayala, et al. 2025), the effect of scraping may have been minimised or masked due to lack of standardisation of scraping time. In the present experiment, precise control of scraping duration revealed that it is this parameter, rather than the number of flushes, that predominantly influences oocyte recovery. It is plausible that increasing the number of flushes after the oocyte has been detached by scraping provides no additional benefit, whereas MF may facilitate oocyte release if scraping is not performed. These results underscore the importance of considering the interaction between technical variables, rather than their isolated effects, when optimising OPU protocols.

In this study, continuous needle rotation for 18 s caused increased operator fatigue, which could negatively impact ORR, as a fatigued operator may not perform scraping effectively (Cuervo‐Arango, Necchi, et al. 2025). While scraping duration is a key determinant of ORR, the optimal rotation time required to achieve acceptable ORR without operator fatigue remains to be established. Furthermore, continuous twisting with SF system appeared to complicate follicle visualisation, which may increase the likelihood of needle displacement compared with the MF system.

The number of system blockages and blood clots observed in the search dish tended to be higher in the SF group, although differences were not statistically significant, likely due to the limited sample size. SF with static fluid during continuous scraping can promote clot formation and system blockages in the long and narrow tubing system, whereas MF allows greater fluid circulation. As described above, the clots adopted a spaghetti‐like morphology. This appearance likely results from prolonged stagnation of blood residues within the circuit in the absence of rinsing, which promotes obstruction at the narrowest points of the system, such as the aspiration needle. In these situations, needle replacement or an open system is often required, potentially affecting the ORR, compromising sterility, and prolonging the overall duration of the procedure.

Interestingly, a higher number of clots did not directly correlate with a lower ORR; however, their presence increased the frequency of blockages and, consequently, the number of system rinses and the total volume of flushing medium used. This finding may explain the absence of significant differences in the volume usage per follicle between the in vivo groups, despite the SF group performing up to nine times fewer flushes. Clots impaired filtering and searching by releasing erythrocytes, which increased medium turbidity. In cases with many clots, operators may spend more time searching and potentially miss oocytes, although it was not demonstrated. Therefore, it is recommended to transfer clots to a separate dish during filtering, as done in this study, to maintain clear visibility.

The main limitation of this study was the small sample size for the in vivo group and the limited number of aspirated follicles. It is possible that the SF method may not be applicable to a commercial OPU system due to the described drawbacks (i.e., likely increase in clot and blockage formation, operator fatigue, and reduced follicle visualisation). A second limitation of the study was the lack of in vitro embryo production results, since the study focused only on the ORR and technical variables associated with the OPU procedure. It is believed that blastocyst production should not be affected by the number of flushes used in each group, since the type of flushing media utilised was the same for both technique groups.

Future research ideas could involve continuous low‐pressure flow to minimise clot formation, as distending the follicle with MF or increasing pressure has been shown not to enhance ORR (Cuervo‐Arango, Sala‐Ayala, et al. 2025; Márquez‐Moya et al. 2025).

## 5 Conclusions

This study demonstrates the impact of efficient follicular scraping on oocyte recovery rate, both in vivo and ex vivo conditions, without the need for multiple flushes. Future studies are needed to define an optimised approach that balances scraping duration with minimal flushing, thereby maximising oocyte recovery rates (ORR), minimising operator fatigue, and reducing system blockages and medium usage.

## Author Contributions

**Adrián Márquez‐Moya:** writing – review and editing, methodology, writing – original draft, investigation, formal analysis, data curation, conceptualisation. **Nerea Carreras‐Vico:** writing – review and editing, methodology, investigation. **Laura Sala‐Ayala:** methodology, investigation. **Rebeca Martínez‐Boví:** writing – review and editing, resources. **Juan Cuervo‐Arango:** writing – review and editing, writing – original draft, supervision, resources, project administration, methodology, investigation, formal analysis, conceptualisation, funding acquisition.

## Funding

This work was supported by Universidad San Pablo – CEU (Grant GIR24‐12).

## Disclosure

*Declaration of generative AI and AI—assisted technologies in the writing process*: While preparing this work, ChatGPT was used as an AI‐assisted tool to refine the language and clarity of our written text. Following its use, we thoroughly reviewed and edited the content to ensure accuracy and appropriateness, taking full responsibility for the final published version of the article.

## Conflicts of Interest

The authors declare no conflicts of interest.

## Data Availability

The data that support the findings of this study are available from the corresponding author upon reasonable request.
